# Comparative Bioequivalence and Safety Evaluation of Ibuprofen/Phenylephrine Hydrochloride Fixed‐Dose Combination Tablets in Healthy Chinese Volunteers

**DOI:** 10.1002/cpdd.1625

**Published:** 2025-11-14

**Authors:** Menghan Ye, Rui Zhang, Jing Wan, Jinping Zhou, Pengpeng Guo, Dianwen Yu, Peixia Li, Yani Liu, Shaojun Shi

**Affiliations:** ^1^ Union Hospital Tongji Medical College Huazhong University of Science and Technology Wuhan People's Republic of China

**Keywords:** bioequivalence, fixed‐dose combination tablet, ibuprofen, phenylephrine, safety

## Abstract

This single‐center, randomized, open‐label bioequivalence program compared two fixed‐dose combination (FDC) tablets containing ibuprofen (200 mg) and phenylephrine hydrochloride (10 mg) from different manufacturers in healthy Chinese adults under fasting and fed conditions. A three‐period, partially replicated crossover design was used for the fasting study and a four‐period, fully replicated crossover design for the fed study. Serial plasma samples were collected up to 16 h post‐dose, and pharmacokinetic parameters included C_max_, AUC_0–t_, and AUC_0–∞_ for both analytes. Bioequivalence was assessed using average bioequivalence (ABE) when the within‐subject standard deviation of the reference was <0.294 and reference‐scaled ABE (RSABE) otherwise. The geometric mean ratios (90% CIs) for C_max_, AUC_0–t_, and AUC_0–∞_ of both ibuprofen and phenylephrine fell within 80%–125% in both nutritional states, with RSABE applied to phenylephrine C_max_ where variability was high. Both products were well tolerated; adverse events were mild, comparable between test and reference, and no subject discontinued due to adverse events. These findings demonstrate bioequivalence of the two ibuprofen/phenylephrine FDC and support their similar safety profiles in healthy Chinese volunteers.

The common cold is a prevalent illness throughout all seasons and is known for its recurring nature.^1^ Despite being a self‐limiting disease, most patients prefer medication for treatment, making anti‐cold drugs a significant segment within the vast domestic pharmaceutical market. These medications are a key segment in pharmaceutical markets. Cold symptoms are usually diverse, and fixed‐dose combinations (FDC) that can address multiple symptoms at once offer the advantage of symptom control, making them mainstream products in the current market. Therefore, effective FDC remain a focal point and hot topic in the development of new cold medications.

The FDC tablet of ibuprofen/phenylephrine hydrochloride [trade name: Advil Congestion Relief (200 mg:10 mg)] is a new type of compound formulation created by substituting phenylephrine hydrochloride (PE) for pseudoephedrine hydrochloride (PSE) found in the commercially available FDC tablet of ibuprofen/pseudoephedrine [trade name: Advil Cold & Sinus (200 mg:30 mg)].^2^ Ibuprofen, a derivative of propionic acid, is rapidly absorbed when ingested orally, reaching peak plasma concentrations within 1–2 h, with a plasma half‐life of about 2 h and 99% binding to plasma proteins.^3^ It slowly penetrates the synovial cavity and maintains a high concentration there, around 90% of the oral dose is excreted in the urine as metabolites. Ibuprofen is an effective PG synthase inhibitor with anti‐inflammatory, antipyretic, and analgesic effects, primarily used for treating rheumatic and rheumatoid arthritis, as well as for general fever reduction and pain relief.^4^ Phenylephrine hydrochloride acts as an α1‐ADR agonist with very weak action on β‐ADR. Its effects are similar to but weaker than norepinephrine. However, due to its stable chemical properties, its action is long lasting, mainly causing constriction of the nasal mucosa blood vessels, thereby treating mucosal congestion during a cold.^5^

Although compound cold medicines composed of antipyretic analgesics and decongestants are available domestically, their decongestant component is mainly pseudoephedrine. Pseudoephedrine's chemical structure is very similar to methamphetamine (meth), making it easy to illicitly convert to the latter.^6^, ^7^ Currently, there are only two types of new anti‐cold drugs in China that do not contain pseudoephedrine, with insufficient clinical supply, inadequate market competition, and impacting public medication accessibility. The FDC tablet of ibuprofen/phenylephrine hydrochloride uses phenylephrine instead of pseudoephedrine, which has anti‐inflammatory and analgesic properties, the dual‐action formulation provides relief from both nasal congestion and pain associated with colds or allergies. There are no pharmacokinetic interactions between the two active ingredients, ibuprofen and phenylephrine, and there are no issues with illegal conversion, making it suitable for a compound formulation and an excellent variety.^8^ Therefore, the current study was designed to compare the bioequivalence and safety profile of two FDC tablets of ibuprofen/phenylephrine hydrochloride (200mg:10 mg) under fasting and fed conditions in healthy Chinese volunteers to seek regulatory approval for the generic formulation to be marketed in China.

## Methods

### Compliance with Ethics Guidelines

This research was conducted under the guidance of the Declaration of Helsinki, Good Clinical Practice (GCP) guidelines of the China Food and Drug Administration (CFDA) and authorized by the independent ethics committee of Tongji Medical College, Huazhong University of Science and Technology ([2021]0992). Written informed consent from each volunteer is required before any procedure can proceed. Clinical trial Registration Numbers: ChiCTR2200057755 and ChiCTR2400091939.

### Study Drug

The test formulation (200 mg ibuprofen and 10 mg phenylephrine hydrochloride; batch number: 200902) was supplied by Honz Pharmaceutical Co., Ltd. The reference formulation (200 mg:10 mg; batch number: R75062) was manufactured by Pfizer. Both products used in this study were obtained from Honz Pharmaceutical Co., Ltd.

### Ethics Approval

This study was conducted at Wuhan Union Hospital, following the GCP guidelines, the Declaration of Helsinki, and all applicable national regulations. The study received ethics committee approval, permitting its execution. Before enrollment, all participants were thoroughly informed about the study objectives, methodology, potential drug effects, expected benefits, possible adverse reactions, and associated risks. Only those who voluntarily agreed to participate provided written informed consent.

### Study Population

Eligible participants were healthy Chinese males and females aged 19 to 50 years (including boundary values) with a body mass index (BMI) between 19 and 26 kg/m^2^. Male subjects had a minimum weight of 50 kg, while female subjects weighed at least 45 kg. Health status was assessed based on demographic data, medical and personal history, recent and current medication use, vital signs (temperature, pulse, blood pressure), physical examination, 12‐lead electrocardiogram (ECG), and laboratory tests, including complete blood count, urinalysis, blood biochemistry, coagulation function, infectious disease screening, pregnancy testing (for females), alcohol breath test, urine drug screening, and tobacco use test. Key exclusion criteria included abnormal medical assessments, history of significant diseases, or hypersensitivity to ibuprofen, phenylephrine, or any excipients. A detailed list of inclusion and exclusion criteria is provided in the Supplemental Information.

### Sample Size Estimation

Sample size estimation for both fasting and fed studies was performed based on the two one‐sided t‐test (TOST) method, taking into account intra‐subject variability derived from pilot data. The pilot data were obtained from an internal pre‐study conducted under fed conditions (n = 6), in which the within‐subject CV for phenylephrine C_max_ was 37.5% and the GMR was 0.95. Accordingly, a three‐period partially replicated design was adopted for the fasting trial and a four‐period fully replicated design for the fed trial to ensure adequate statistical power.

For the fasting trial, the highest coefficient of variation (CV) for C_max_ was 29.56% for phenylephrine. With a significance level of 0.05 and a power of 80%, the minimum sample size required was 31 subjects. To compensate for an expected 20% dropout rate, 39 healthy subjects were enrolled. For the fed trial, the CV for phenylephrine C_max_ was 43.5%, leading to a calculated sample size of 34. Accordingly, 40 subjects were enrolled to ensure sufficient statistical power. These estimations followed standard regulatory guidelines for bioequivalence evaluation.

### Study Design

The study was composed of two separate parts: one was a single‐center, randomized, open‐label, three‐period, and reference‐replicated crossover study under fasting conditions and the other was a single‐center, randomized, open‐label, four‐period, and fully replicated crossover study under fed conditions. The primary objective of this study was to evaluate the equivalence of the test drug and reference drug in Chinese healthy subjects under fasting and fed conditions. The secondary objective was to evaluate the safety of the two drugs.

The estimated sample size was 39 subjects in fasting trial and 40 subjects in fed trial. In the fed trial, subjects were required to consume a high‐fat, high‐calorie meal 30 min before drug administration. A standardized meal (∼900 kcal: ∼150 kcal protein, ∼250 kcal carbohydrates, and ∼500 kcal fat) was provided, consisting of whole milk, eggs, bread, and butter. Fasting subjects were randomly divided into three groups (Group A, Group B, and Group C) at a ratio of 1:1:1, all of which were orally administered in the fasting state. Each group had three periods, and one pill was given on the first day of each period. The washout period was 7 days. Fed subjects were randomly assigned 1:1 to TRTR and RTRT, both of which were administered orally in the fed state, where T represents acceptance of the test preparation and R represents acceptance of the reference preparation. Subjects in each group received a single dose of T or R each period, one tablet each time, under fed conditions. The washout period was 7 days.

### Bioanalytical Method Validation

The bioanalytical method was validated according to current regulatory guidelines (FDA and EMA) prior to sample analysis. Validation parameters included selectivity, linearity, accuracy, precision, recovery, matrix effect, and stability under short‐term and long‐term storage conditions.

Calibration curves demonstrated excellent linearity over 100–25,000 ng/mL for ibuprofen and 0.0200–5.00 ng/mL for phenylephrine, with correlation coefficients (R^2^) > 0.99. Intra‐day precision (%CV) ranged from 4.1 to 5.0 for ibuprofen and 4.9 to 8.8 for phenylephrine, while accuracy (%bias) ranged from −1.4 to −0.4 for ibuprofen and −4.7 to −1.9 for phenylephrine. Mean extraction recovery was 92%–105%, and no significant matrix effect or carryover was observed. All results met the regulatory acceptance limits (±15%, ±20% for LLOQ), confirming method reliability.

### PK Analysis

For both fasting and fed conditions, blood samples for pharmacokinetic (PK) analysis were collected at pre‐dose (within 1 h) and at 22 time points post‐administration, including 10 min, 20 min, 30 min, 45 min, 1 h, 1.25 h, 1.5 h, 1.75 h, 2 h, 2.25 h, 2.5 h, 3 h, 3.5 h, 4 h, 4.5 h, 5 h, 6 h, 8 h, 10 h, 12 h, and 16 h. This included a blank blood sample. In total, 66 blood samples were collected per subject under fasting conditions, while 88 blood samples were obtained per subject under fed conditions.

At each time point, 5 mL of venous blood was drawn into K_2_‐EDTA anticoagulant tubes and immediately placed in ice water to maintain sample integrity. Primary PK parameters included C_max_, AUC_0–t_, and AUC_0–∞_, while secondary PK parameters encompassed T_max_, t_1_/_2_, λz, AUC_%Extrap, F, and others. Plasma concentrations of ibuprofen and phenylephrine were determined by a validated LC–MS/MS method as described in the Bioanalytical Method Validation section.

### Bioequivalence Analysis

Given the high variability of phenylephrine hydrochloride, bioequivalence assessments were conducted using two approaches based on the intra‐subject variability of the reference formulation. When the within‐subject standard deviation (SWR) of the reference formulation was ≥0.294 (equivalent to CV ≥ 30%), the reference‐scaled average bioequivalence (RSABE) method was applied in accordance with FDA guidance for highly variable drugs. Under this approach, bioequivalence was concluded when the 95% upper confidence bound of the linearized RSABE criterion was ≤0, indicating that the variability‐adjusted comparison met regulatory equivalence requirements. In addition, the geometric mean ratios (GMRs) of C_max_ and AUC parameters needed to fall within the standard bioequivalence range of 80.00%–125.00%. If SWR < 0.294, the ABE method was used, and bioequivalence was established when the 90% confidence intervals of the GMRs for C_max_, AUC_0–t_, and AUC_0–∞_ were within 80.00%–125.00%.

### Safety Analysis

Any clinically significant abnormalities observed relative to baseline were classified as adverse events (AEs). Safety assessments included vital signs, physical examinations, 12‐lead ECG, laboratory tests, adverse events, adverse reactions, and serious adverse events (SAEs).

Adverse events were analyzed using the safety set (SS), with all reported AEs during the treatment period coded according to the Medical Dictionary for Regulatory Activities (MedDRA, version 25.0). The severity and incidence of AEs were categorized based on System Organ Class (SOC) and Preferred Term (PT) classifications within MedDRA.

### Statistical Analysis

In this study, pharmacokinetic (PK) analysis of key parameters was conducted using Phoenix WinNonlin (version 8.3) with a non‐compartmental model. Additional statistical analyses were performed using SAS 9.4, and related databases were exported and stored in SAS XPORT format.

The statistical analysis plan (SAP) was developed by the biostatistician and the principal investigator in accordance with the study protocol, refined as necessary, and finalized prior to data locking. The analysis methods included: (1)Descriptive statistics for quantitative data, presented as mean, standard deviation, median, quartiles, minimum, maximum, and coefficient of variation. (2) Categorical and ordinal data, summarized as frequency and percentage. (3) Bioequivalence assessment, with a 90% confidence interval (CI) applied to the ABE test. (4) No interim or subgroup analyses were planned. However, in cases where the exclusion of subjects was disputed and had potential impact on the results, a sensitivity analysis was conducted to assess the robustness of the conclusions.

## Results

### Subjects

**Figure 1:**
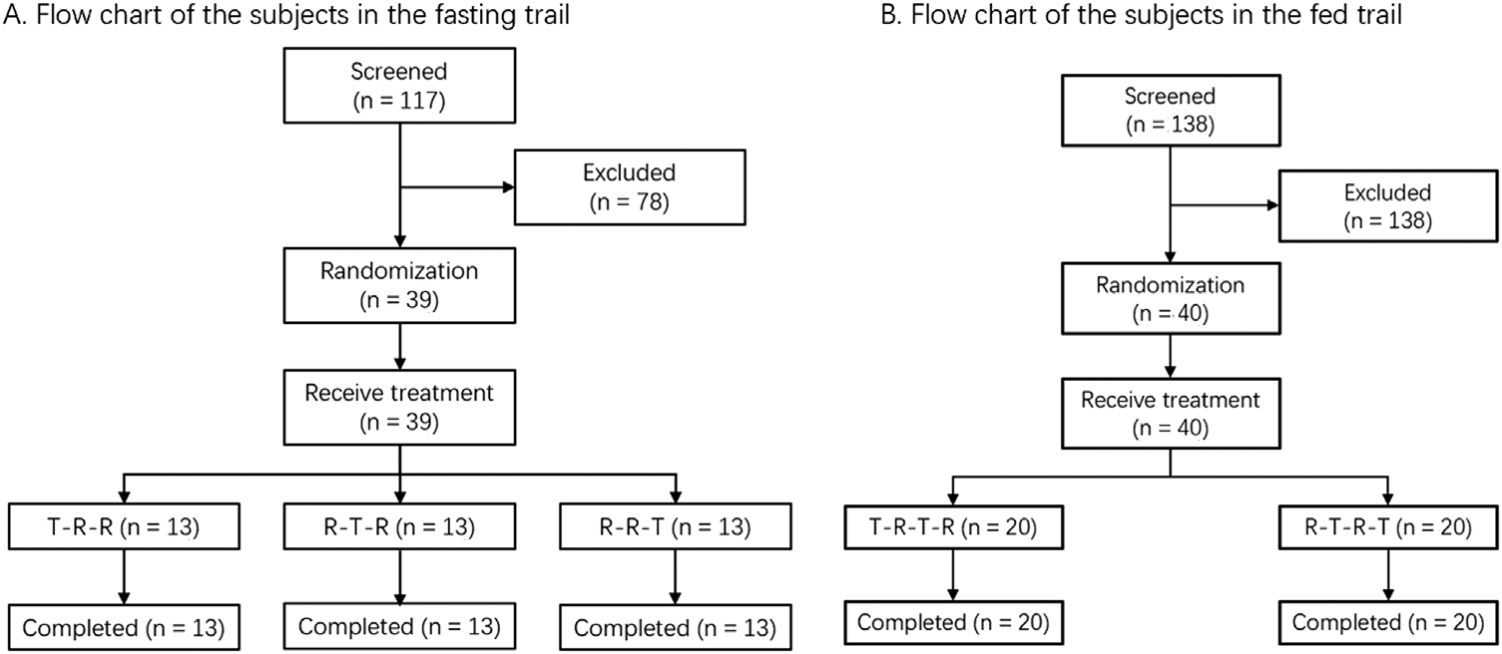
Participant disposition in the fasting (n = 39) and fed (n = 40) studies. The diagram summarizes screening, enrollment, randomization to treatment sequences, period completions, and analysis sets for each nutritional state.

A total of 117 healthy volunteers were screened for the fasting trial. According to the inclusion and exclusion criteria, 39 volunteers were invited for the fasting trial (Figure 1a). Among the 138 potential healthy volunteers in the fed trial, 40 volunteers were finally enrolled in the trial (Figure 1b). There were no significant differences in the subject demographics and baseline characteristics between the fasting and fed trials, respectively (*P* > .05) (Table. 1). Furthermore, baseline demographic parameters were well balanced across treatment sequences in both studies, with no statistically significant differences in age, height, weight, or BMI among sequence groups (*P* > .05). Detailed sequence‐wise demographic data are provided in Tables S1 and S2.

**Table 1:** Demographic Baseline.

	Fasting N = 39	Fed N = 40
**Age (years)**		
N (N miss)	39 (0)	40 (0)
Mean (SD)	28.9 (6.38)	29.5 (6.39)
Median (Q1, Q3)	29.0 (23.0, 34.0)	28.0 (25.0, 34.5)
Min, Max	(19, 42)	(20, 43)
**Height (cm)**		
N (N miss)	39 (0)	40 (0)
Mean (SD)	165.81 (8.203)	167.65 (8.182)
Median (Q1, Q3)	166.50 (159.50, 173.50)	167.75 (163.25, 173.25)
Min, Max	(147.0, 177.5)	(151.0, 188.5)
**Weight (kg)**		
N (N miss)	39 (0)	40 (0)
Mean (SD)	60.76 (7.328)	62.80 (7.952)
Median (Q1, Q3)	61.10 (55.10, 66.10)	62.75 (57.05, 67.15)
Min, Max	(46.5, 77.4)	(48.4, 81.3)
**BMI (kg/m^2^)**		
N (N miss)	39 (0)	40 (0)
Mean (SD)	21.99 (1.398)	22.25 (1.758)
Median (Q1, Q3)	21.90 (21.00, 22.60)	22.00 (20.85, 23.25)
Min, Max	(19.3, 25.6)	(19.5, 25.9)
**Age stratification, n (%)**		
N (N miss)	39 (0)	40 (0)
18–40	37 (94.9)	38 (95.0)
41–64	2 (5.1)	2 (5.0)
65–75	0 (0)	0 (0)
>75	0 (0)	0 (0)
**Gender, n (%)**		
N (N miss)	39 (0)	40 (0)
Male	25 (64.1)	25 (62.5)
Female	14 (35.9)	15 (37.5)
**Nation, n (%)**		
N (N miss)	39 (0)	40 (0)
Ethnic Han	36 (92.3)	37 (92.5)
Other nationalities	3 (7.7)	3 (7.5)

BMl, body mass index; n, number of subjects; SD, standard deviation.

### Pharmacokinetics

**Figure 2:**
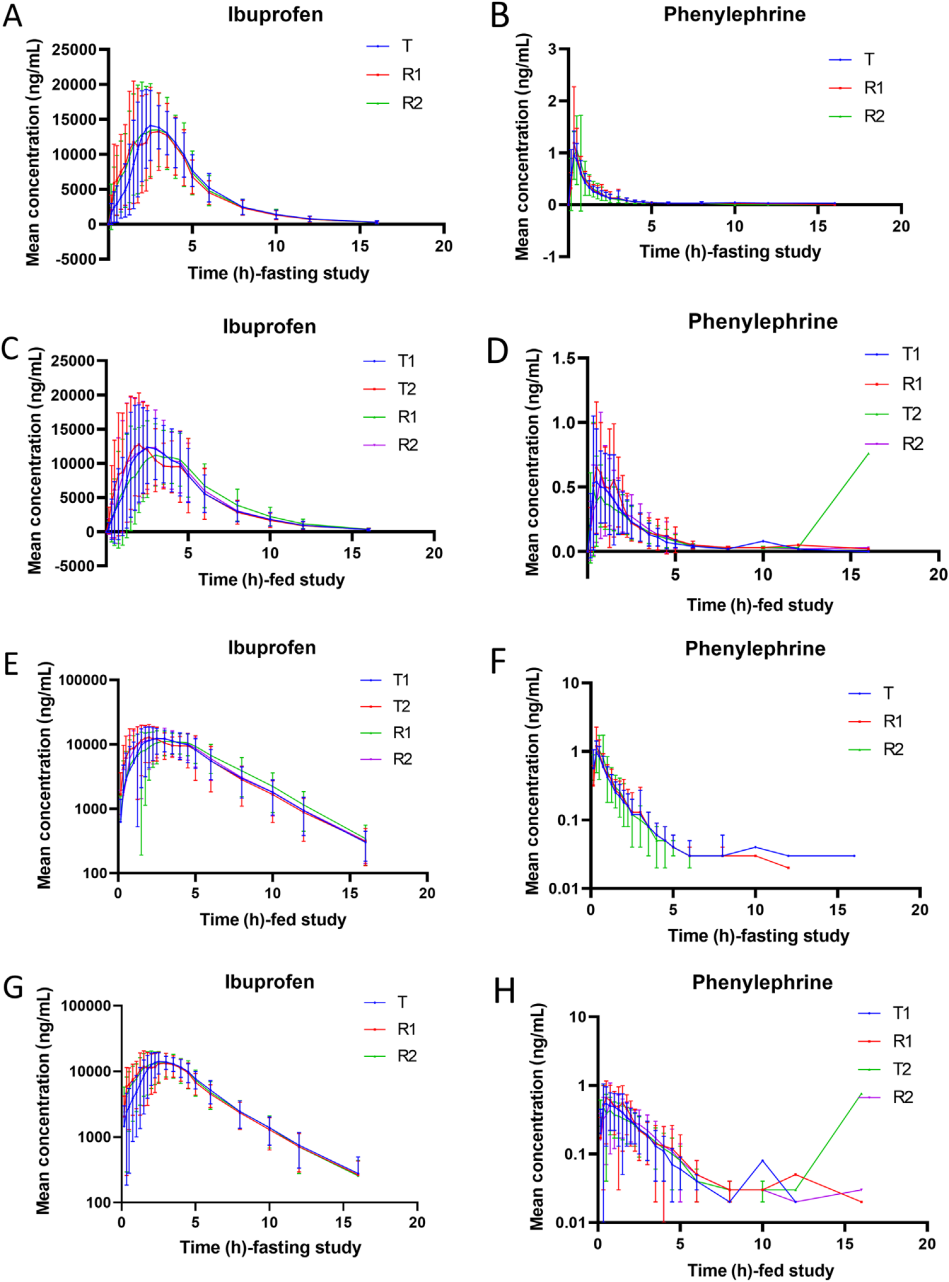
(A) Average concentration–time (C–T) curve of ibuprofen in the fasting trial; (B) Average concentration–time (C–T) curve of phenylephrine in the fasting trial; (C) Average concentration–time (C–T) curve of ibuprofen in the fed trial; (D) Average concentration–time (C–T) curve of phenylephrine in the fed trial; (E) Semilog concentration–time curve of ibuprofen in the fasting trial; (F) Semilog concentration–time curve of phenylephrine in the fasting trial; (G) Semilog concentration–time curve of ibuprofen in the fed trial; (H) Semilog concentration–time curve of phenylephrine in the fed trial. Error bars represent standard error of the mean (SEM).

The mean (± SD) plasma concentration–time (C–T) curves of ibuprofen and phenylephrine hydrochloride following single‐dose oral administration of three individual sequence (T1, R1, and R2) under fasting conditions and four individual sequence (T1, T2, R1, and R2) under fed conditions are shown in Figure 2. The primary PK parameters under fasting and fed conditions are summarized in Tables 2 and 3, with additional geometric mean and GCV% data provided in Tables S3 and S4.

**Table 2:** Ibuprofen and Phenylephrine Pharmacokinetic Parameters Were Administered in the Fed Trial.

	T1 (n = 40))		T2 (n = 40)		R1 (n = 40)		R2 (n = 40)	
	Mean (SD)	%CV	Mean (SD)	%CV	Mean (SD)	%CV	Mean (SD)	%CV
**Ibuprofen**								
T_max_ (h)*	2.13 (0.49, 5.00)	48.27	3.00 (0.75, 8.00)	51.22	1.99 (0.49, 5.00)	58.44	2.38 (0.75, 5.00)	48.36
C_max_ (ng/mL)	17,810.50 (4230.12)	23.75	16,657.25 (4124.25)	24.76	19,767.50 (4666.27)	23.61	19,352.50 (4882.75)	25.23
AUC_0–t_ (ng h/mL)	68,707.43 (16,436.13)	23.92	71,105.22 (16,603.66)	23.35	69,003.88 (16,750.01)	24.27	70,854.88 (16,473.07)	23.25
AUC_0–∞_ (ng h/mL)	69,663.31 (16,779.00)	24.09	72,202.44 (17,187.12)	23.80	70,017.29 (17,149.93)	24.49	71,895.09 (16,943.99)	23.57
λ_z_ (per h)	0.33 (0.04)	13.29	0.33 (0.05)	15.84	0.32 (0.05)	15.11	0.32 (0.05)	15.38
t_1/2_ (h)	2.17 (0.29)	13.26	2.17 (0.31)	14.48	2.20 (0.34)	15.67	2.22 (0.33)	14.98
AUC__%Extrap_ (%)	1.34 (0.52)	38.98	1.42 (0.73)	51.45	1.41 (0.74)	52.29	1.39 (0.64)	46.39
**Phenylephrine**								
T_max_ (h)*	1.00 (0.33, 3.00)	67.00	1.25 (0.16, 16.00)	141.45	1.25 (0.33, 4.50)	80.79	1.50 (0.33, 4.50)	59.04
C_max_ (ng/mL)	0.79 (0.47)	59.22	0.66 (0.36)	53.99	0.92 (0.50)	54.36	0.72 (0.36)	50.13
AUC_0–t_ (ng h/mL)	1.22 (0.27)	22.41	1.18 (0.37)	31.28	1.28 (0.33)	25.72	1.12 (0.28)	25.38
AUC_0–∞_ (ng h/mL)	1.29 (0.30)	23.38	1.22 (0.26)	21.49	1.35 (0.35)	26.22	1.20 (0.29)	24.37
λ_z_ (per h)	0.56 (0.22)	38.70	0.54 (0.24)	45.11	0.63 (0.27)	42.51	0.60 (0.24)	39.22
t_1/2_ (h)	1.51 (0.86)	57.01	1.76 (1.36)	77.42	1.38 (0.94)	68.22	1.51 (1.17)	77.34
AUC__%Extrap_ (%)	4.93 (3.25)	65.80	6.10 (3.79)	62.09	4.94 (3.97)	80.31	5.69 (3.85)	67.75

λ_z_, terminal rate constant in the plasma; AUC__%Extrap_, the percentage of the area under the concentration–time curve that is calculated by extrapolation beyond the last measured point; AUC_0–∞_, the total area under the concentration–time curve from drug administration extrapolated to infinity, indicating overall drug exposure; AUC_0–t_, the AUC of the analyte in the plasma over the time interval from time zero to the last measurable concentration; C_max_, the maximum observed drug concentration in the plasma; t_1/2_, the terminal half‐life of the analyte in the plasma; T_max_, the time from administration to the maximum observed concentration of the analyte in the plasma (expressed as median and range, indicated by “*”).

**Table 3:** Ibuprofen and Phenylephrine Pharmacokinetic Parameters Were Administered in the Fasting Trial.

	T (n = 39)		R1 (n = 39)		R2 (n = 39)	
	Mean (SD)	%CV	Mean (SD)	%CV	Mean (SD)	%CV
**Ibuprofen**						
T_max_ (h)*	2.25 (1.50,4.50)	32.44	2.50 (0.33,4.50)	43.04	2.25 (0.75,4.50)	43.98
C_max_ (ng/mL)	17,989.74 (3131.15)	17.41	21,194.87 (5083.15)	23.98	21,058.97 (4624.75)	21.96
AUC_0–t_ (ng h/mL)	68,903.36 (15,109.73)	21.93	69,634.56 (15,851.09)	22.76	71,422.35 (15,903.69)	22.27
AUC_0–∞_ (ng h/mL)	69,941.98 (15,859.11)	22.67	70,566.29 (16,206.60)	22.97	72,309.35 (16,503.09)	22.82
λ_z_ (per h)	0.30 (0.05)	16.59	0.31 (0.04)	14.45	0.31 (0.04)	12.41
t_1/2_ (h)	2.35 (0.42)	17.91	2.28 (0.34)	14.88	2.24 (0.30)	13.37
AUC__%Extrap_ (%)	1.37 (0.96)	70.00	1.29 (0.77)	59.63	1.16 (0.62)	53.30
**Phenylephrine**						
T_max_ (h)*	0.33 (0.16,3.00)	95.13	0.33 (0.33,3.00)	93.36	0.49 (0.16,2.00)	67.86
C_max_ (ng/mL)	1.10 (0.36)	33.02	1.34 (0.97)	72.05	1.31 (0.90)	68.66
AUC_0–t_ (ng h/mL)	1.18 (0.31)	26.27	1.24 (0.37)	30.09	1.28 (0.55)	43.17
AUC_0–∞_ (ng h/mL)	1.24 (0.32)	26.23	1.32 (0.37)	27.75	1.34 (0.56)	41.74
λ_z_ (per h)	0.61 (0.27)	44.41	0.55 (0.27)	48.83	0.58 (0.24)	41.61
t_1/2_ (h)	1.49 (0.95)	63.79	1.68 (1.23)	72.93	1.45 (0.74)	50.68
AUC__%Extrap_ (%)	4.67 (2.34)	49.97	5.61 (5.05)	90.00	4.69 (2.56)	54.62

λ_z_, terminal rate constant in the plasma; AUC__%Extrap_, the percentage of the area under the concentration–time curve that is calculated by extrapolation beyond the last measured point; AUC_0–∞_, the total area under the concentration–time curve from drug administration extrapolated to infinity, indicating overall drug exposure; AUC_0–t_, the AUC of the analyte in the plasma over the time interval from time zero to the last measurable concentration; C_max_, the maximum observed drug concentration in the plasma; t_1/2_, the terminal half‐life of the analyte in the plasma; T_max_, the time from administration to the maximum observed concentration of the analyte in the plasma (expressed as median and range, indicated by “*”).

### Bioequivalence

The bioequivalence evaluation between the test and reference FDC tablets of ibuprofen/phenylephrine hydrochloride in healthy volunteers under fasting and fed conditions is presented in Tables 4 and 5.

**Table 4:** Results of Bioequivalence Evaluation of Major Pharmacokinetic Parameters of Ibuprofen and Phenylephrine in the Fasting Trial.

PK Parameter (Unit)	T/R Point Estimation (%)	SWR	CV_W_ (%)	90% Confidence Interval (ABE)	95% Upper Confidence (RSABE)	Evaluation Method	Result
**Ibuprofen**							
C_max_ (ng/mL)	86.08	0.17	17.59	(82.17, 90.17)	0.02	ABE	Bioequivalent
AUC_0–t_ (ng h/mL)	97.69	0.08	8.34	(95.30, 100.15)	−0.00	ABE	
AUC_0–∞_ (ng h/mL)	97.84	0.08	8.18	(95.38, 100.36)	−0.00	ABE	
**Phenylephrine**							
C_max_ (ng/mL)	90.03	0.42	43.89	(83.16, 97.48)	−0.08	RSABE	Bioequivalent
AUC_0–t_ (ng h/mL)	94.91	0.16	16.25	(91.87, 98.05)	−0.01	ABE	
AUC_0−∞_ (ng h/mL)	94.37	0.16	16.44	(91.20, 97.64)	−0.01	ABE	

ABE, average bioequivalence; AUC_0–∞_, the total area under the concentration–time curve from drug administration extrapolated to infinity, indicating overall drug exposure; AUC_0–t_, the AUC of the analyte in the plasma over the time interval from time zero to the last measurable concentration; C_max_, the maximum observed drug concentration in the plasma; CVw, within‐subject coefficient of variation of reference drug; PK, pharmacokinetic; RSABE, reference scale average bioequivalence; SWR, intra‐individual standard deviation of the reference preparation.

**Table 5:** Results of Bioequivalence Evaluation of Major Pharmacokinetic Parameters of Ibuprofen and Phenylephrine in the Fed Trial.

PK Parameter (Unit)	T/R Point Estimation (%)	SWR	CV_W_ (%)	90% Confidence Interval (ABE)	95% Upper Confidence (×10^−2^ RSABE)	Evaluation Method	Result
**Ibuprofen**							
C_max_ (ng/mL)	88.22	0.15	15.33	(84.44, 92.16)	1.11	ABE	Bioequivalent
AUC_0–t_ (ng h/mL)	100.01	0.08	7.80	(98.32, 101.72)	−0.35	ABE	
AUC_0–∞_ (ng h/mL)	99.99	0.08	7.69	(98.30, 101.69)	−0.34	ABE	
**Phenylephrine**							
C_max_ (ng/mL)	87.71	0.35	36.02	(79.71, 96.51)	−3.72	RSABE	Bioequivalent
AUC_0–t_ (ng h/mL)	100.00	0.11	10.97	(96.59, 103.54)	−0.68	ABE	
AUC_0–∞_ (ng h/mL)	99.59	0.11	10.67	(96.29, 102.84)	−0.61	ABE	

ABE, average bioequivalence; AUC_0–∞_, the total area under the concentration–time curve from drug administration extrapolated to infinity, indicating overall drug exposure; AUC_0–t_, the AUC of the analyte in the plasma over the time interval from time zero to the last measurable concentration; C_max_, the maximum observed drug concentration in the plasma; CVw, within‐subject coefficient of variation of reference drug; PK, pharmacokinetic; RSABE, reference scale average bioequivalence; SWR, intra‐individual standard deviation of the reference preparation.

Thirty‐nine fasting subjects were included in the analysis. For ibuprofen, the SWR values of C_max_, AUC_0–t_, and AUC_0–∞_ were 0.17, 0.08, and 0.08, respectively—all below the 0.294 threshold. Therefore, the ABE method was applied. The GMRs and 90% confidence intervals (CIs) for C_max_, AUC_0–t_, and AUC_0–∞_ were 86.08% (82.17%, 90.17%), 97.69% (95.30%, 100.15%), and 97.84% (95.38%, 100.36%), respectively, all within the 80.00%–125.00% range. For phenylephrine hydrochloride, the SWR values of C_max_, AUC_0–t_, and AUC_0–∞_ were 0.42, 0.16, and 0.16, respectively. As SWR < 0.294 for AUC parameters, ABE was applied, while RSABE was used for C_max_ due to high variability. The GMRs (90% CIs) for C_max_, AUC_0–t_, and AUC_0–∞_ were 90.03% (83.16%, 97.48%), 94.91% (91.87%, 98.05%), and 94.37% (91.20%, 97.64%), respectively, confirming bioequivalence.

Forty subjects were included in the fed trial. The SWR values for ibuprofen were 0.15 (C_max_), 0.08 (AUC_0–t_), and 0.08 (AUC_0–∞_), all <0.294. Thus, ABE was used, and the GMRs (90% CIs) were 88.22% (84.44%, 92.16%), 100.01% (98.32%, 101.72%), and 99.99% (98.30%, 101.69%), respectively. For phenylephrine hydrochloride, SWR values were 0.35 (C_max_), 0.11 (AUC_0–t_), and 0.11 (AUC_0–∞_). ABE was used for AUC parameters, and RSABE was applied for C_max_. The GMRs (90% CIs) for C_max_, AUC_0–t_, and AUC_0–∞_ were 87.71% (79.71%, 96.51%), 100.00% (96.59%, 103.54%), and 99.59% (96.29%, 102.95%), respectively, supporting bioequivalence.

### Safety Results

During the fasting trial, a total of 12 subjects had 20 adverse events after administration, and the overall incidence of adverse events was 30.8%. During the single administration of test preparation T, five subjects (12.8%) had eight adverse events; no adverse events occurred during the first administration of reference preparation R. During the second administration of reference preparation R, 12 adverse events occurred in 8 subjects (20.5% incidence). Sixteen adverse events were probably related to the study drug, and four adverse events were probably unrelated to the study drug. During the fed experiment, a total of 17 subjects had 20 adverse events after administration, with an overall adverse event rate of 42.5%. During the first administration of test preparation T, two subjects (incidence 5.0%) had two adverse events; Three adverse events occurred in three subjects (7.5%) during the first administration of reference preparation R; during the second administration of test preparation T, seven subjects (17.5%) had seven adverse events; During the second administration of reference preparation R, eight subjects (20.0% incidence) experienced eight adverse events. Of these, 18 cases were probably related to the study drug. In the fasting and feeding trials, the incidence of adverse events was similar for subjects taking test preparation T and reference preparation R each time. All adverse events that occurred in the trial were mild and no action was taken. No participants withdrew from the trial due to adverse events (Tables 6 and 7).

**Table 6:** Summary of AEs in the Fasting Trial.

	T n (%), n′	R1 n (%), n′	R2 n (%), n′	Total n (%), n′
**AEs**	5 (12.8), 8	0 (0), 0	8 (20.5), 12	12 (30.8), 20
**SAEs**	0 (0), 0	0 (0), 0	0 (0), 0	0 (0), 0
**AEs leading to withdrawal**	0 (0), 0	0 (0), 0	0 (0), 0	0 (0), 0
**Laboratory examination**	2 (5.1), 3	0 (0), 0	7 (17.9), 11	9 (23.1), 14
UOB+	0 (0), 0	0 (0), 0	2 (5.1), 2	2 (5.1), 2
Tachycardia	0 (0), 0	0 (0), 0	2 (5.1), 2	2 (5.1), 2
Hypertriglyceridemia	0 (0), 0	0 (0), 0	2 (5.1), 2	2 (5.1), 2
Leukocytosis	0 (0), 0	0 (0), 0	1 (2.6), 1	1 (2.6), 1
High LDL	1 (2.6), 1	0 (0), 0	0 (0), 0	1 (2.6), 1
Hematuria+	0 (0), 0	0 (0), 0	1 (2.6), 1	1 (2.6), 1
Basophilia	0 (0), 0	0 (0), 0	1 (2.6), 1	1 (2.6), 1
Eosinophilia if elevated	0 (0), 0	0 (0), 0	1 (2.6), 1	1 (2.6), 1
Hypercholesterolemia	1 (2.6), 1	0 (0), 0	0 (0), 0	1 (2.6), 1
Hypokalemia	1(2.6), 1	0(0), 0	0(0), 0	1(2.6), 1
Neutrophilia	0(0), 0	0(0), 0	1(2.6), 1	1(2.6), 1
**Systemic disease**	1(2.6), 1	0(0), 0	0(0), 0	1(2.6), 1
Chest Pain	1(2.6), 1	0(0), 0	0(0), 0	1(2.6), 1
**The gastrointestinal system**	2(5.1), 2	0(0), 0	1(2.6), 1	3(7.7), 3
Nausea	0(0), 0	0(0), 0	1(2.6), 1	1(2.6), 1
Vomiting	2(5.1), 2	0(0), 0	0(0), 0	2(5.1), 2
**Neurological disorders**	2(5.1), 2	0(0), 0	0(0), 0	2(5.1), 2
Headache	2(5.1), 2	0(0), 0	0(0), 0	2(5.1), 2

AE, adverse events; n (%), the number of subjects experiencing the adverse event and the corresponding incidence rate; n′, the number of occurrences (event counts) of the adverse event; SAE, serious adverse events.

**Table 7:** Summary of AEs in the Fed Trial.

	T1 n (%), n′	T2 n (%), n′	R1 n (%), n′	R2 n (%), n′	Total n (%), n′
**AEs**	2 (5.0), 2	7 (17.5), 7	3 (7.5), 3	8 (20.0), 8	17 (42.5), 20
**SAEs**	0 (0), 0	0 (0), 0	0 (0), 0	0 (0), 0	0 (0), 0
**AEs leading to withdrawal**	0 (0), 0	0 (0), 0	0 (0), 0	0 (0), 0	0 (0), 0
**Laboratory examination**	1 (2.5), 1	3 (7.5), 3	3 (7.5), 3	8 (20.0), 8	14 (35.0), 15
Tachycardia	1 (2.5), 1	0 (0), 0	3 (7.5), 3	1 (2.5), 1	4 (10.0), 5
Anemia	0 (0), 0	1 (2.5), 1	0 (0), 0	2 (5.0), 2	3 (7.5), 3
Abnormal QRS axis	0 (0), 0	0 (0), 0	0 (0), 0	1 (2.5), 1	1 (2.5), 1
Positive urinary leukocytes	0 (0), 0	0 (0), 0	0 (0), 0	1 (2.5), 1	1 (2.5), 1
Electrocardiogram ST‐T segment changes	0 (0), 0	0 (0), 0	0 (0), 0	1 (2.5), 1	1 (2.5), 1
ECG abnormalities	0 (0), 0	1 (2.5), 1	0 (0), 0	0 (0), 0	1 (2.5), 1
Hyperbilirubinemia	0 (0), 0	0 (0), 0	0 (0), 0	1 (2.5), 1	1 (2.5), 1
Hypertriglyceridemia	0 (0), 0	0 (0), 0	0 (0), 0	1 (2.5), 1	1 (2.5), 1
Hyperuricemia	0 (0), 0	1 (2.5), 1	0 (0), 0	0 (0), 0	1 (2.5), 1
**Neurological disorders**	1 (2.5), 1	2 (5.0), 2	0 (0), 0	0 (0), 0	2 (5.0), 3
Dizziness	1 (2.5), 1	2 (5.0), 2	0 (0), 0	0 (0), 0	2 (5.0), 3
**Heart disease**	0 (0), 0	2 (5.0), 2	0 (0), 0	0 (0), 0	2 (5.0), 2
Intraventricular conduction delay	0 (0), 0	2 (5.0), 2	0 (0), 0	0 (0), 0	2 (5.0), 2

AE, adverse events; n (%), the number of subjects experiencing the adverse event and the corresponding incidence rate; n′, the number of occurrences (event counts) of the adverse event; SAE, serious adverse events.

## Discussion

As a standard Phase Ⅰ clinical bioequivalence trial, preference is usually given to healthy male and female volunteers to avoid the potential influence of disease factors and drug interference. In order to fully assess the effect of food on drug absorption and metabolism, and to ensure the safety and efficacy of the drug in different physiological states, the equivalence evaluation test of this product should be conducted in both fasting and fed tests.

We conducted a pilot study under fed conditions to estimate within‐subject variability and guide the formal bioequivalence study design. The pre‐study adopted a two‐period complete replicated crossover design in healthy Chinese subjects and showed a relatively high intra‐subject variability for phenylephrine (CV = 37.5%) with a GMR of 0.95. These findings were used to determine the sample size and replication model for the pivotal trials.

Considering the characteristics of the ibuprofen/phenylephrine FDC and the variability of phenylephrine pharmacokinetics, a two‐formulation, three‐period, partially replicated crossover design was employed under fasting conditions, while a two‐formulation, four‐period, fully replicated design was used under fed conditions. The three‐period design efficiently evaluates bioavailability under minimal food interference, whereas the four‐period design better accounts for the variability introduced by food effects. This combined approach enhances statistical power, improves intra‐subject consistency, and ensures a comprehensive assessment of bioequivalence under different physiological states.

The pharmacokinetic characteristics observed in this study were generally consistent with those reported in recent investigations of ibuprofen and phenylephrine, supporting the validity of our bioequivalence findings. For ibuprofen, both fasting and fed parameters (C_max_ ≈ 25–30 µg/mL; T_max_ ≈ 1–1.5 h) closely matched those reported in healthy Chinese volunteers, where food delayed absorption and slightly reduced C_max_ without affecting overall exposure.^9^ Similar results have been described in population pharmacokinetic analyses and recent bioequivalence trials, which demonstrated that food primarily prolongs the absorption phase but has minimal influence on total systemic exposure.^10^

Phenylephrine, a selective α_1_‐adrenergic agonist, showed rapid absorption (T_max_ ≈ 1 h) and a short half‐life (≈ 1.5 h), in line with previous studies (C_max_ 0.9–1.6 ng/mL; T_max_ 0.3–1.2 h).^8^ The food‐induced delay in peak concentration but unchanged total exposure observed here mirrors findings from controlled studies in both adult and pediatric populations.^11^ The high intra‐subject variability of phenylephrine is well recognized and largely attributable to extensive first‐pass metabolism by monoamine oxidase and catechol‐O‐methyltransferase,^2^ which can lead to variable systemic availability. These consistent results across different studies and formulations confirm that the pharmacokinetic behaviors of both ibuprofen and phenylephrine in our study align with established absorption and metabolic patterns, reinforcing the robustness and generalizability of our bioequivalence conclusions.

Based on FDA guidelines, ABE and RSABE are the two basic methods for determining whether two formulations are bioequivalent.^12^ Therefore, ABE method and RSABE method were used in this study to establish the BE of the tested drug and the reference drug for the main pharmacokinetic parameters. According to our results, the two primary components, ibuprofen and phenylephrine hydrochloride in two FDC products met the equivalence criteria. However, subjects in the fasting trial were different from those in the fed trial, so any statistical differences between before and after meals were not analyzed

At the clinical trial stage, there was no significant safety difference between the generic drug and the original drug. During the trial, a total of 29 subjects experienced 40 adverse events after administration, all of which were mild adverse events, of which 34 cases were possibly related to administration. All adverse events were eliminated or stabilized, and no subjects withdrew from the trial due to adverse events. The results showed that ibuprofen and its generics had good safety. Because clinical trials are conducted under a variety of different conditions, the adverse reaction rate observed in a clinical trial of one drug cannot be directly compared with the adverse reaction rate observed in a clinical trial of another drug, and may not reflect the actual situation.

This study was conducted at a single center with a moderate sample size, which, while sufficient for bioequivalence evaluation, may limit the generalizability of the results. Furthermore, all enrolled subjects were healthy Chinese adults. Potential racial or ethnic differences in drug metabolism, absorption, and enzyme activity may affect the pharmacokinetic performance of the FDC in broader populations. These factors should be considered when interpreting the findings, and future multicenter studies involving more diverse populations are warranted.

## Conclusion

This is a Phase I clinical trial of the FDC tablets of ibuprofen/hydrochloride phenylephrine and its generic tablets in healthy Chinese subjects. These trial data confirm that two FDC tablets have bioequivalence and good safety in healthy Chinese subjects. The results achieved the expected goal and provided support for the clinical application of the FDC tablets of ibuprofen/hydrochloride phenylephrine in China.

## Author Contributions

Menghan Ye was responsible for writing the original draft. Rui Zhang contributed to review and editing, methodology development, and project administration. Jing Wan and Peixia Li performed the experimental investigation. Jinping Zhou carried out the formal analysis. Pengpeng Guo was responsible for validation. Dianwen Yu designed the visualizations. Yani Liu provided supervision. Shaojun Shi conceived the study and secured funding.

## Funding

This work was supported by National Natural Science Foundation of China (82474008, 82173902, and 82104274).

## Conflicts of Interest

The authors declare no conflicts of interest.

## Supporting information



Supplemental Information: The Supplemental Information contain the detailed exclusion criteria for the study population. These criteria include subject history, medication usage restrictions, lifestyle factors, and specific laboratory test thresholds that determined eligibility for participation. The full exclusion criteria are provided in the supplementary file titled Supplementary.docx.

## Data Availability

The datasets used and analyzed during the current study available from the corresponding author on reasonable request.
