# Supplementary material for: Comparative Bioequivalence and Safety Evaluation of Ibuprofen/Phenylephrine Hydrochloride Fixed‐Dose Combination Tablets in Healthy Chinese Volunteers
Source: Clin Pharmacol Drug Dev. 2025 Nov 14;15(3):e1625. doi: 10.1002/cpdd.1625 (PMC12946568; doi:10.1002/cpdd.1625)
Supplement: Supplementary file 1 — Supplemental Information: The Supplemental Information contain the detailed exclusion criteria for the study population. These criteria include subject history, medication usage restrictions, lifestyle factors, and specific laboratory test thresholds that determined eligibility for participation. The full exclusion criteria are provided in the supplementary file titled Supplementary.docx. [file CPDD-15-0-s001.docx]

# Exclusion criteria

1. (Inquiry) Subjects with a history of allergy to ibuprofen, oxymetazoline, or their excipients (microcrystalline cellulose, cross-linked sodium carboxymethyl cellulose, colloidal silicon dioxide, hydroxypropyl methylcellulose, sodium lauryl sulfate, talc, magnesium stearate, enteric-coated film-forming agents, etc.), or individuals with a history of allergy to two or more drugs, food, etc;
2. (Inquiry) Subjects with a history of swallowing difficulties or any gastrointestinal diseases that may affect drug absorption;
3. (Inquiry) Subjects with a history of mental or neurological disorders or a family history of hereditary diseases such as epilepsy or depression;
4. (Inquiry) Subjects with a history of surgery, trauma, or planned surgery during the study period that may affect the safety of the trial or the pharmacokinetics of the drug;
5. (Inquiry) Subjects who have taken any medication, health supplements, or traditional Chinese medicine within 2 weeks prior to screening;
6. (Inquiry) Subjects who have taken monoamine oxidase inhibitors (such as phenelzine, isocarboxazid, moclobemide, selegiline, brompheniramine, tolcapone, etc.) within 1 month prior to screening;
7. (Inquiry) Subjects who have taken illicit drugs within 3 months prior to screening;
8. (Inquiry) Subjects with a history of drug abuse within 6 months prior to screening;
9. (Inquiry) Subjects who smoke more than 5 cigarettes per day in the past 3 months or cannot stop using any tobacco products during the trial;
10. (Inquiry) Subjects with a weekly alcohol consumption exceeding 14 units (1 unit = 17.7 mL of ethanol, i.e., 1 unit = 357 mL of beer with 5% alcohol content or 43 mL of Baijiu with 40% alcohol content or 147 mL of wine with 12% alcohol content), or individuals who cannot abstain from alcohol during the trial;
11. (Inquiry) Subjects who have consumed excessive amounts of tea, coffee, and/or caffeinated beverages (more than 8 cups, 1 cup = 250 mL) per day in the past 3 months or cannot discontinue their consumption during the trial;
12. (Inquiry) Subjects who have participated in other drug clinical trials within 3 months prior to administration or who are not participating in clinical trials themselves;
13. (Inquiry) Subjects who have donated blood, including component blood or experienced significant blood loss (≥400 mL), received blood transfusion or used blood products within 3 months prior to screening, or are planning to donate blood during the trial;
14. (Inquiry) Subjects who have received vaccination within 1 month prior to screening, or are planning to be vaccinated during the study period or within 1 month after the end of the study.
15. (Inquiry) Female subjects who are breastfeeding;
16. (Inquiry) Subjects and their partners who have pregnancy plans or plans to donate sperm or eggs during the trial period (from screening to 6 months after the end of the trial) and who are unwilling or unable to voluntarily adopt effective contraception measures;
17. (Inquiry) Subjects who cannot tolerate venipuncture or have a history of needle or blood phobia;
18. (Inquiry) Subjects who are lactose intolerant or fructose intolerant (have experienced diarrhea after drinking milk);
19. (Inquiry) Subjects with special dietary requirements or who cannot accept a standardized diet;
20. Subjects with positive urine drug screening for opioids, ketamine, methamphetamine, MDMA, or tetrahydrocannabinol acid (THCA), or positive results in a tobacco test;
21. Subjects with abnormal results with clinical significance in vital signs examination, physical examination, clinical laboratory tests (complete blood count, urinalysis, blood biochemistry, infectious disease screening, coagulation function), and 12-lead electrocardiogram;
22. Subjects with alcohol breath test result greater than 0.0 mg/100 mL;
23. Female subjects of childbearing age with positive pregnancy test results during the screening period or trial process;
24. Subjects with acute illness occurring before the use of investigational drugs;
25. Subjects with positive results in the nucleic acid test for novel coronavirus;
26. Subjects who may not be able to complete the study for other reasons or whom the investigator deems should not be included.

**Table S1.** Demographic characteristics by treatment sequence in the fed trial.

|  | TRTR (N=20) | RTRT (N=20) | Total (N=40) |
| --- | --- | --- | --- |
| **Age (years)** |  |  |  |
| N (missing) | 20 (0) | 20 (0) | 40 (0) |
| Mean (SD) | 29.1 (6.86) | 30.0 (6.02) | 29.5 (6.39) |
| Median (Q1, Q3) | 26.5 (24.5, 36.0) | 29.0 (25.0, 33.0) | 28.0 (25.0, 34.5) |
| Min, Max | (20, 43) | (21, 43) | (20, 43) |
| **Height (cm)** |  |  |  |
| N (missing) | 20 (0) | 20 (0) | 40 (0) |
| Mean (SD) | 166.70 (8.682) | 168.61 (7.755) | 167.65 (8.182) |
| Median (Q1, Q3) | 166.75 (159.50, 174.00) | 168.25 (164.25, 172.30) | 167.75 (163.25, 173.25) |
| Min, Max | (151.0, 181.0) | (152.0, 188.5) | (151.0, 188.5) |
| **Body Weight (kg)** |  |  |  |
| N (missing) | 20 (0) | 20 (0) | 40 (0) |
| Mean (SD) | 61.82 (7.847) | 63.78 (8.136) | 62.80 (7.952) |
| Median (Q1, Q3) | 61.70 (56.00, 65.15) | 63.60 (59.40, 68.55) | 62.75 (57.05, 67.15) |
| Min, Max | (51.0, 81.3) | (48.4, 76.9) | (48.4, 81.3) |
| **BMI (kg/m²)** |  |  |  |
| N (missing) | 20 (0) | 20 (0) | 40 (0) |
| Mean (SD) | 22.16 (1.747) | 22.33 (1.810) | 22.25 (1.758) |
| Median (Q1, Q3) | 21.90 (20.85, 22.85) | 22.15 (20.75, 23.90) | 22.00 (20.85, 23.25) |
| Min, Max | (19.9, 25.9) | (19.5, 25.6) | (19.5, 25.9) |
| **Age stratification, n(%)** | |  |  |
| N (missing) | 20 (0) | 20 (0) | 40 (0) |
| 18–40 | 19 (95.0) | 19 (95.0) | 38 (95.0) |
| 41–64 | 1 (5.0) | 1 (5.0) | 2 (5.0) |
| 65–75 | 0 (0) | 0 (0) | 0 (0) |
| >75 | 0 (0) | 0 (0) | 0 (0) |
| **Gender, n(%)** |  |  |  |
| N (missing) | 20 (0) | 20 (0) | 40 (0) |
| Male | 12 (60.0) | 13 (65.0) | 25 (62.5) |
| Female | 8 (40.0) | 7 (35.0) | 15 (37.5) |
| **Nation, n(%)** |  |  |  |
| N (missing) | 20 (0) | 20 (0) | 40 (0) |
| Ethnic Han | 18 (90.0) | 19 (95.0) | 37 (92.5) |
| Other | 2 (10.0) | 1 (5.0) | 3 (7.5) |

**Note:** T denotes the test formulation and R denotes the reference formulation. TRR indicates that subjects received the test formulation in Period 1, the reference formulation in Period 2, and the reference formulation again in Period 3; RTR indicates that subjects received the reference formulation in Period 1, the test formulation in Period 2, and the reference formulation in Period 3; RRT indicates that subjects received the reference formulation in Periods 1 and 2, and the test formulation in Period 3. The percentage in the table was calculated as (number of subjects in the corresponding subgroup / total number of subjects in each sequence) × 100%.

**Table S2.** Demographic characteristics by treatment sequence in the fasting trial.

|  | TRR (N=13) | RTR (N=13) | RRT (N=13) | Total (N=39) |
| --- | --- | --- | --- | --- |
| **Age (years)** |  |  |  |  |
| N (missing) | 13 (0) | 13 (0) | 13 (0) | 39 (0) |
| Mean (SD) | 29.9 (8.10) | 27.9 (5.33) | 28.8 (5.73) | 28.9 (6.38) |
| Median (Q1, Q3) | 32.0 (22.0, 36.0) | 28.0 (24.0, 31.0) | 29.0 (25.0, 33.0) | 29.0 (23.0, 34.0) |
| Min, Max | (21, 42) | (19, 36) | (21, 40) | (19, 42) |
| **Height (cm)** |  |  |  |  |
| N (missing) | 13 (0) | 13 (0) | 13 (0) | 39 (0) |
| Mean (SD) | 164.69 (6.909) | 166.73 (7.846) | 166.00 (10.073) | 165.81 (8.203) |
| Median (Q1, Q3) | 162.50 (159.50, 170.00) | 166.50 (163.50, 170.00) | 170.50 (154.50, 174.50) | 166.50 (159.50, 173.50) |
| Min, Max | (155.0, 177.5) | (147.0, 177.5) | (150.5, 176.5) | (147.0, 177.5) |
| **Body Weight (kg)** |  |  |  |  |
| N (missing) | 13 (0) | 13 (0) | 13 (0) | 39 (0) |
| Mean (SD) | 58.87 (6.300) | 61.18 (7.280) | 62.24 (8.420) | 60.76 (7.328) |
| Median (Q1, Q3) | 60.80 (52.80, 63.10) | 60.40 (56.00, 65.60) | 65.60 (56.90, 67.60) | 61.10 (55.10, 66.10) |
| Min, Max | (46.5, 68.0) | (51.4, 77.4) | (47.7, 75.4) | (46.5, 77.4) |
| **BMI (kg/m²)** |  |  |  |  |
| N (missing) | 13 (0) | 13 (0) | 13 (0) | 39 (0) |
| Mean (SD) | 21.62 (1.433) | 21.89 (1.349) | 22.45 (1.387) | 21.99 (1.398) |
| Median (Q1, Q3) | 21.10 (20.80, 22.00) | 21.70 (20.90, 22.60) | 22.30 (21.50, 23.30) | 21.90 (21.00, 22.60) |
| Min, Max | (19.3, 24.8) | (20.3, 24.5) | (21.0, 25.6) | (19.3, 25.6) |
| **Age stratification, n(%)** | |  |  |  |
| N (missing) | 13 (0) | 13 (0) | 13 (0) | 39 (0) |
| 18–40 | 11 (84.6) | 13 (100) | 13 (100) | 37 (94.9) |
| 41–64 | 2 (15.4) | 0 (0) | 0 (0) | 2 (5.1) |
| 65–75 | 0 (0) | 0 (0) | 0 (0) | 0 (0) |
| >75 | 0 (0) | 0 (0) | 0 (0) | 0 (0) |
| **Gender, n(%)** |  |  |  |  |
| N (missing) | 13 (0) | 13 (0) | 13 (0) | 39 (0) |
| Male | 8 (61.5) | 9 (69.2) | 8 (61.5) | 25 (64.1) |
| Female | 5 (38.5) | 4 (30.8) | 5 (38.5) | 14 (35.9) |
| **Nation, n(%)** |  |  |  |  |
| N (missing) | 13 (0) | 13 (0) | 13 (0) | 39 (0) |
| Ethnic Han | 11 (84.6) | 12 (92.3) | 13 (100) | 36 (92.3) |
| Other | 2 (15.4) | 1 (7.7) | 0 (0) | 3 (7.7) |

**Note:** T denotes the test formulation and R denotes the reference formulation. TRR indicates that subjects received the test formulation in Period 1, the reference formulation in Period 2, and the reference formulation again in Period 3; RTR indicates that subjects received the reference formulation in Period 1, the test formulation in Period 2, and the reference formulation in Period 3; RRT indicates that subjects received the reference formulation in Periods 1 and 2, and the test formulation in Period 3. The percentage in the table was calculated as (number of subjects in the corresponding subgroup / total number of subjects in each sequence) × 100%.

**Table S3.** Ibuprofen and Phenylephrine pharmacokinetic parameters were administered in the fed trial

|  | T1(n=40)) | | | T2(n=40) | | | R1(n=40) | | R2 (n=40) | |
| --- | --- | --- | --- | --- | --- | --- | --- | --- | --- | --- |
|  | Geomean | GCV(%) | Geomean | | GCV(%) | | Geomean | GCV(%) | Geomean | GCV(%) |
| **Ibuprofen** |  |  | |  | |  |  |  |  |  |
| C_max_ (ng/mL) | 17291.93 | 25.87 | | 16152.35 | | 25.87 | 19203.18 | 25.87 | 18689.79 | 29.5 |
| AUC_0-t_ (ng·h/mL) | 68785.9 | 24.71 | | 69216.72 | | 24.71 | 66986.30 | 25.87 | 69002.53 | 23.58 |
| AUC_0-∞_ (ng·h/mL) | 67691.34 | 24.71 | | 70217.41 | | 24.71 | 67946.09 | 25.87 | 69974.79 | 24.71 |
| **Phenylephrine** | |  | |  | |  |  |  |  |  |
| C_max_ (ng/mL) | 0.69 | 51.91 | | 0.59 | | 0.48 | 0.81 | 0.51 | 0.65 | 45.64 |
| AUC_0-t_ (ng·h/mL) | 1.19 | 22.48 | | 1.14 | | 0.26 | 1.24 | 25.87 | 1.09 | 24.71 |
| AUC_0-∞_ (ng·h/mL) | 1.26 | 22.48 | | 1.19 | | 21.41 | 1.31 | 27.06 | 1.17 | 23.58 |

**Notes:** C_max_: the maximum observed drug concentration in the plasma; AUC_0-t_: the AUC of the analyte in the plasma over the time interval from time zero to the last measurable concentration;AUC_0-∞_: The total area under the concentration-time curve from drug administration extrapolated to infinity, indicating overall drug exposure. Geomean: geometric mean; GCV: geometric coefficient of variation.

**Table S4.** Ibuprofen and Phenylephrine pharmacokinetic parameters were administered in the fasting trial.

|  | T(n=39) | | R1(n=39) | | R2 (n=39) | |
| --- | --- | --- | --- | --- | --- | --- |
|  | Geomean | GCV(%) | Geomean | GCV(%) | Geomean | GCV(%) |
| **Ibuprofen** |  |  |  |  |  |  |
| C_max_ (ng/mL) | 17719.64 | 18.47 | 20603.55 | 24.71 | 20566.65 | 22.48 |
| AUC_0-t_ (ng·h/mL) | 67424.41 | 21.41 | 68123.86 | 21.41 | 69918.40 | 20.23 |
| AUC_0-∞_ (ng·h/mL) | 68362.58 | 21.41 | 69018.00 | 21.41 | 70738.56 | 21.41 |
| **Phenylephrine** |  |  |  |  |  |  |
| C_max_ (ng/mL) | 1.04 | 35.44 | 1.15 | 54.84 | 1.16 | 45.64 |
| AUC_0-t_ (ng·h/mL) | 1.14 | 28.02 | 1.19 | 28.02 | 1.21 | 34.34 |
| AUC_0-∞_ (ng·h/mL) | 1.20 | 28.02 | 1.28 | 24.71 | 1.27 | 34.34 |

**Notes:** C_max_, the maximum observed drug concentration in the plasma; AUC_0-t_, the AUC of the analyte in the plasma over the time interval from time zero to the last measurable concentration; AUC_0-∞_, The total area under the concentration-time curve from drug administration extrapolated to infinity, indicating overall drug exposure. Geomean: geometric mean; GCV: geometric coefficient of variation.
